# Diabetic Bone Disease and Diabetic Myopathy: Manifestations of the Impaired Muscle-Bone Unit in Type 1 Diabetes

**DOI:** 10.1155/2022/2650342

**Published:** 2022-05-12

**Authors:** Callie Travis, Priya S. Srivastava, Thomas J. Hawke, Evangelia Kalaitzoglou

**Affiliations:** ^1^University of Kentucky College of Medicine, Lexington, KY, USA; ^2^Department of Pediatrics, Division of Pediatric Endocrinology, UCSF Benioff Children's Hospital, San Francisco, CA, USA; ^3^Department of Pathology and Molecular Medicine, McMaster University, Hamilton, ON, Canada; ^4^University of Kentucky, Barnstable Brown Diabetes Center, Lexington, KY, USA; ^5^Department of Pediatrics, University of Kentucky College of Medicine, Lexington, KY 40536, USA

## Abstract

Type 1 diabetes is associated with complications affecting muscle and bone, with diabetic bone disease and diabetic myopathy becoming increasingly reported in the past few decades. This review is aimed at succinctly reviewing the literature on the current knowledge regarding these increasingly identified and possibly interconnected complications on the musculoskeletal system. Furthermore, this review summarizes several nonmechanical factors that could be mediating the development and progression of premature musculoskeletal decline in this population and discusses preventative measures to reduce the burden of diabetes on the musculoskeletal system.

## 1. Introduction

Type 1 diabetes (T1D) is a life-long disease diagnosed primarily during childhood. It is characterized by autoimmune destruction of the *β*-cells within the pancreas, resulting in insulin deficiency and dependence on exogenous insulin. Despite those with T1D striving to maintain normal glycemic control, less than a third of patients consistently achieve target blood glucose levels [1]. Therefore, glycemic variability and complications of diabetes represent a considerable aspect of the disease. Furthermore, there is a significant increase in the prevalence of T1D. Specifically, between 2001 and 2009, there was a 21 percent increase in people diagnosed with T1D under the age of 20, with the prevalence of T1D increasing not only in the United States [2] but also worldwide [3]. Because of the aforementioned facts, T1D-associated complications and the associated costs are expected to rise.

Traditionally, T1D has been associated with macrovascular and microvascular complications, such as retinopathy, neuropathy, and diabetic kidney disease. Less traditionally recognized complications of T1D include diabetic bone disease (DBD) and diabetic myopathy. Clinically, DBD has increasingly gained attention in recent years as a complication of T1D with multiple studies reporting low bone mineral density and increased incidence of fracture in patients with T1D compared to the general population [4, 5]. Awareness of T1D-associated myopathy has also increased in recent years with reduced muscle mass and strength, as well as altered metabolic capacities being reported [6–10].

**Figure 1:**
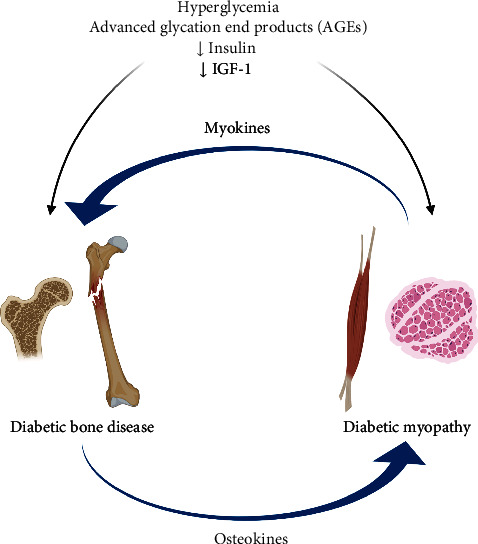
Common underlying mechanisms of diabetic bone disease and diabetic myopathy in type 1 diabetes (created with http://BioRender.com/).

Skeletal muscle and bone are two organs that develop concurrently and are in close proximity. Bone mass and microscopic geometry follow the development of body size and muscle force in children and adolescents [11]. In fact, adolescence and young adulthood are times of significant muscle and bone accrual. The muscle-bone unit has been described as an operational unit in which the mechanical loading of bone is dependent on muscle strength [11]. Potential nonmechanical interactions between muscle and bone, including muscle-derived myokines, bone-derived osteokines, and other common underlying mechanisms in T1D could explain why the muscle-bone unit appears to be affected soon after diagnosis and throughout the duration of the disease (Figure 1).

### 1.1. Diabetic Bone Disease in Humans with T1D

T1D-associated DBD manifests itself as decreased bone mineral density, higher risk of fracture, abnormal bone microstructure, and low bone turnover [5]. Several studies have shown that individuals with T1D have lower bone mineral density [12–14], with the cortical area being inversely associated with long-term glycemic control [14]. Although most studies have shown decreased bone mineral density as assessed by dual-energy X-ray absorptiometry (DXA), a limited number of studies have reported deficits in 3-D bone microarchitecture by high-resolution peripheral quantitative computed tomography (HR-pQCT) or magnetic resonance imaging (MRI). Specifically, Shanbhogue et al. [15] reported deficits in bone properties in those with T1D, particularly in the presence of microvascular complications. These deficits were primarily in the trabecular compartment (decreased number of trabeculae and trabecular thickness) and are similar to those observed in conditions of increased bone turnover [15]. Furthermore, trabecular microarchitecture assessed by MRI at the tibia has also been shown to be impaired (reduced bone volume and trabecular number and increased trabecular separation) in young women with T1D with onset during childhood, compared to healthy young women [16].

The risk for fracture in T1D is present at all ages and for all sites. A population-based study from The Health Improvement Network (THIN) in the UK reported an increased incidence of any fracture in individuals with T1D starting from childhood [17]. Adolescents with T1D have decreased bone mass and size [13, 18] and are at risk of growth failure with lower mean height SDS [19] and relative resistance to growth hormone [20]. Recent studies have confirmed decreased bone mineral density coupled with deficits in skeletal muscle properties of children and adolescents with T1D [21, 22].

Overall, the risk for fracture is present in both sexes based on a recent meta-analysis, which reported that women with T1D have four times the risk and men have two times the risk of experiencing a bone fracture when compared to the general population [23]. This significantly higher risk of fracture could be due to multiple factors contributing to bone health in individuals with T1D. Age, longer duration of T1D, and T1D diagnosis before peak bone mass is achieved are considered important risk factors for fracture [24].

In addition to deficits in bone mass, bone turnover is decreased in T1D, with most studies revealing a decrease in bone formation and bone resorption [25]. Specifically, a study comparing children with T1D to age-matched healthy children showed lower osteocalcin, procollagen type-1 amino-terminal propeptide (PINP), and type 1 collagen C-terminal cross-linking telopeptide (CTX) levels in children with T1D [26]. Additionally, in young adult women with T1D, CTX levels have also been found to be lower than age-matched healthy women [16].

The Iowa Women's Health Study showed that postmenopausal women with T1D had 12.25-fold higher risk of hip fracture compared to women without T1D. Overall, the risk of fracture has been considered to be higher in women with T1D compared to men, according to a recent meta-analysis [23]. Other studies in patients with T1D have reported similar cumulative probability of getting a hip fracture in both sexes before age 40, but thereafter, increasing more rapidly among men [27]. Based on these findings, it appears that female sex could play a role in risk of fracture in T1D, although men with T1D should also be considered to be at high risk for fracture.

### 1.2. Diabetic Myopathy in Humans with T1D

Loss of skeletal muscle mass and decline in muscle function is a characteristic of aging and usually takes several decades to progress. It is most pronounced in the 7th and 8th decades of life. However, individuals with T1D begin to exhibit diabetes-associated muscle decline at a much younger age [28]. Indeed, a small study involving newly diagnosed young individuals with insulin-dependent diabetes showed diffuse muscle fiber atrophy compared to age-matched controls [29]. Additionally, children with T1D and poor glycemic control exhibit lower hand-grip strength compared to age-matched controls [22]. Another study in adolescents with T1D, divided into groups based on the duration of diabetes, concluded that there was a decrease in the relative muscle power and force in adolescent participants with a longer duration of T1D [21]. A very recent study in adults showed that maximal contraction demonstrated a faster decline after 35 years of age in otherwise healthy adults with T1D. This was accompanied by more prominent type 1 myofiber grouping and an increased incidence of hybrid myofibers (fibers expressing more than one myosin heavy chain isoform) compared to healthy controls [6]. The results from the above studies support the presence of accelerated skeletal muscle aging in patients with T1D [28].

The presence of muscle atrophy [30] and decreased muscle strength [31–33] in those with T1D is expected in those diagnosed with neuropathy. Muscle biopsies from patients with T1D of various duration showed negative effects on skeletal muscle ultrastructure, which was more pronounced in the presence of diabetic neuropathy [34]. Another study by Orlando et al. found impaired muscle strength and fatigability in participants with T1D, which was more pronounced in the group with polyneuropathy. This study concluded that factors other than nerve damage contribute to the impaired muscle phenotype associated with T1D [35]. However, although historically myopathy was considered a result of neuropathy in T1D, recent studies have provided strong evidence that those with T1D exhibit muscle-specific alterations, even in the absence of neurologic complications [6, 7]. A possible underlying mechanism for the muscle fatigability seen in young adults with T1D without complications could be impaired mitochondrial oxidative capacity, a finding that was recently reported by Monaco et al. [7].

Further study of those with T1D showed differences between men and women in skeletal muscle mitochondrial bioenergetics, which plays a key role in basic cellular processes, including but not limited to metabolism and cell survival [10]. Because of the mechanical stress that muscle plays on bone development and maintenance, these findings suggest that premature skeletal muscle aging negatively affects bone quality, and thus leads to higher risks of fracture, particularly in females with T1D.

### 1.3. Potential Nonmechanical Drivers of Muscle-Bone Unit Dysfunction in T1D

#### 1.3.1. Trophic Factors

Insulin and insulin-like growth factor (IGF-1) are anabolic factors for bone and skeletal muscle. Specifically, insulin stimulates growth factors involved in myogenesis [36], and it plays a critical role in skeletal muscle protein synthesis and breakdown [37]. In the skeleton, it is considered to have anabolic effects [38], and it promotes osteoblast differentiation [39]. IGF-1 is required for longitudinal bone growth, skeletal maturation, and bone mass acquisition not only during growth but also in the maintenance of bone throughout life [40]. IGF-1 is also essential for skeletal myogenesis and is associated with muscle mass, muscle development, and regeneration. Furthermore, it increases the proliferative capacity of muscle satellite cells (MSCs) [41].

T1D is a disease associated with low systemic insulin and IGF-1 levels in humans [16, 20, 42]. Individuals with T1D frequently exhibit insulin resistance, even in adolescence [43–45], which has been associated with increased risk of micro- and macrovascular complications [43, 46]. Furthermore, markers of insulin resistance and *β*-cell function have been found to be inversely associated with muscle strength in young adults [47]. Additionally, trabecular bone quality in those with T1D has been shown to be negatively associated with insulin resistance [48] and, in adolescent boys, insulin resistance negatively affects bone development [49].

Lack of endogenous insulin production and reduced insulin and IGF-1 signaling due to resistance could be a contributing factor to the decrease in bone formation, as well as muscle deficits and impaired muscle repair that are observed in T1D.

#### 1.3.2. Hyperglycemia and Advanced Glycation End Products (AGEs)

Hyperglycemia and the resulting advanced glycation end products (AGE) have also been implicated in the poor bone microarchitecture that predisposes to fracture. Poor glycemic control has been associated with bone deficits in many studies [22, 50, 51]. In iliac crest bone biopsies from patients with T1D, bone pentosidine, a type of AGE, was higher in patients with T1D who had a history of fragility fractures than those without fractures or healthy controls [52]. Furthermore, in a study of adult patients with T1D, high serum pentosidine was found to be associated with prevalent fractures [53]. In a Japanese cohort of patients with T1D, subcutaneous AGE accumulation was associated with impaired lower limb skeletal muscle function [54]. Additionally, children with T1D in poor glycemic control have reduced aerobic muscle capacity [55] and reduced muscle area [22]. These studies suggest that hyperglycemia and the resulting AGEs are drivers of impairment of the muscle-bone unit in T1D and could significantly contribute to DBD and diabetic myopathy of T1D.

#### 1.3.3. Osteokines and Myokines

Signaling between bone and muscle can be divided into two categories: bone-to-muscle signaling and muscle-to-bone signaling. Bone-derived signaling molecules (osteokines) include osteocalcin, sclerostin, FGF-23, Wnt-3a, and TGF-*β* [56]. Osteocalcin is a bone-derived hormone secreted primarily by osteoblasts. Some studies have reported that its levels are reduced in T1D [57], particularly in children and adolescents [58–60], although some studies show no change in osteocalcin levels with T1D. In addition to its role in bone mineralization, osteocalcin is important in free fatty acid and glucose uptake [61] and mitochondrial biogenesis and insulin sensitivity [62] in skeletal muscle.

Sclerostin, a negative regulator of bone formation, has been found to be higher in children with T1D, [63, 64]. It is unclear whether higher levels of sclerostin are associated with poor bone outcomes, although one of these studies showed higher sclerostin levels associated with impaired mineral status in children [64]. Additionally, sclerostin has been implicated in muscle homeostasis [65]. However, this has not been studied in patients with T1D. FGF23 levels do not seem to differ between those with and without T1D [66]. The role of other osteokines, such as TGF-*β*, in musculoskeletal health in humans with T1D is not known. Additional studies are needed to evaluate the role of osteokines in DBD and myopathy.

Muscle-to-bone signaling molecules (myokines) include myostatin, interleukins (IL-6, IL-7, and IL-15), IGF-1, FGF-2, and irisin. Myostatin is a myokine secreted primarily by skeletal muscle [67]. It negatively regulates muscle mass [67] and has been shown to negatively affect osteoblastogenesis [68]. Myostatin has been found to be elevated systemically in adults with T1D, although its levels have not been shown to be elevated in skeletal muscle of those with T1D [69]. Contrary to myostatin, irisin is a myokine that positively regulates muscle. It is secreted by skeletal muscle following exercise resulting in increased glucose and fatty acid uptake [70], and it induces mitochondrial biogenesis and thermogenesis [71]. In bone, irisin has positive effects on osteoblast differentiation and mineralization [72]. Higher systemic levels of irisin have been shown to correlate with better glycemic control and relate to bone parameters in children with T1D [73] as well as with lower insulin requirements in women with T1D [74]. Another study found lower irisin levels in those with T1D compared to healthy controls [75]. Irisin has been shown to be pro-osteogenic therefore, lower irisin levels could account for poor bone parameters in T1D.

Interleukin-6 can be derived from the muscle. IL-6 was assessed in patients with T1D by Rachon et al., and it was noted that postmenopausal women with T1D had significantly lower femoral neck BMD and an increased serum bioactive IL-6 level compared to control groups. However, after the use of multiple regression studies, no firm correlation was made between the two parameters [76]. Interleukin-15 has also been found to be elevated in young adults with T1D [77]. However, it is not clear whether this is associated with any deficits in bone or skeletal muscle. Further studies are needed to explore the role of myokines in DBD and diabetic myopathy.

### 1.4. Prevention and Management of DBD and Diabetic Myopathy

Despite the increasing prevalence of T1D and the associated poor bone and skeletal muscle health, there has been limited research and no consensus on the prevention and treatment of DBD and myopathy [78]. In order to improve bone fragility and declining muscle function in this population, it is important to understand how to identify those at risk as well as possible interventions with various treatments. Longer disease duration, chronic poor glycemic control, low body mass index, and the presence of diabetic complications have been identified as risk factors for DBD [78]. Multiple studies have noted that bone mineral density T-score and FRAX can be used as screening tools for risk of fracture in patients with diabetes, but there is often underestimation of risk in this population that must be adjusted [79]. Lifestyle interventions, such as physical activity, and optimization of glycemic control and dietary factors (calcium and vitamin D supplementation) have been recommended as possible interventions to mitigate the risk for fracture.

First, lifestyle adjustments like weight-bearing exercise have not been studied extensively in relation to bone and muscle parameters in patients with T1D. However, in one study evaluating the effects of exercise in children with T1D, it was noted that regular weight-bearing exercise for 9 months three times per week improved total body and lumbar spine bone mineral density [26]. Another study also showed improvements in bone density with a 3-month exercise program in adolescents with T1D [80]. Exercise has also been shown to improve maximum strength in individuals with T1D [81] and also improve glycemic control and muscle health [82]. Exercise can improve insulin sensitivity, decrease insulin requirements, decrease microvascular complications, and reduce cardiovascular disease in patients with T1D [83], although it can be associated with exercise-induced hypoglycemia. This risk can be mitigated with use of continuous glucose monitors and mealtime insulin dose adjustments depending on the duration, intensity, and type of physical activity [84]. Thus, weight-bearing exercise should be recommended for patients with T1D due to the aforementioned reasons and, potentially, as a means to decrease the risk for fracture and improve skeletal muscle function and strength.

Second, some studies support that patients with T1D have suboptimal calcium intake and lower 25 (OH)- D levels. Specifically, a recent cross-sectional study by Weber found that one third of youth with T1D studied had inadequate calcium intake [85]. Another study from Brazil reported low dietary calcium intake in 76% of children with T1D and optimal serum vitamin D levels in only 18% of children with T1D [86]. In that same study, calcium intake was correlated with bone mass. Furthermore, a meta-analysis reported lower serum vitamin D levels in patients with T1D compared to healthy controls [87]. Careful monitoring of vitamin D status and calcium intake as well as supplementation, if indicated, should be considered in this population, although there is only scarce evidence that vitamin D and calcium supplementation are associated with better musculoskeletal outcomes in T1D [88].

Finally, some studies support that tight glycemic control is inversely related to risk of fracture, while both hypoglycemia and hyperglycemia are directly related to risk of fracture. In a study by Neumann et al. involving men and premenopausal women with T1D, long-term glycemic control was associated with increased clinical fracture prevalence but not with bone mineral density [89]. Another study reported that premenopausal women with T1D and good glycemic control when compared to nondiabetic women had no decrease in bone density in the phalangeal bones, however, premenopausal women with T1D and poor glycemic control had decreased formation and increased resorption of bone [90]. In contrast to these studies, a study looking at vertebral fractures did not find an association with glycemic control [91]. Furthermore, hyperglycemia and resulting AGEs have been associated with reduced muscle strength and muscle mass in T1D [92], and poor diabetes control has been found to impair muscle oxygen supply during exercise in T1D [93], indicating that tight glycemic control would be beneficial for skeletal muscle properties in T1D.

## 2. Conclusions

DBD and diabetic myopathy are becoming increasingly recognized complications of T1D. Because skeletal muscle and bone develop in proximity, determinants of skeletal muscle health can affect the health of bone and vice versa. When assessing bone and skeletal muscle deficits in T1D, one should consider common drivers, such as insulin deficiency, hyperglycemia, osteokines, and myokines that could contribute to both the impairments in skeletal muscle and bone seen in this disease. Lifestyle interventions, such as exercise, supplementation with calcium/vitamin D, and tight glycemic control could provide some protection and improve musculoskeletal health in T1D.
